# Fish Is Food - The FAO’s Fish Price Index

**DOI:** 10.1371/journal.pone.0036731

**Published:** 2012-05-08

**Authors:** Sigbjørn Tveterås, Frank Asche, Marc F. Bellemare, Martin D. Smith, Atle G. Guttormsen, Audun Lem, Kristin Lien, Stefania Vannuccini

**Affiliations:** 1 CENTRUM Católica Graduate School of Business, Pontificia Universidad Católica del Perú, Lima, Peru; 2 Department of Industrial Economics, University of Stavanger, Stavanger, Norway; 3 Sanford School of Public Policy, Duke University, Durham, North Carolina, United States of America; 4 Department of Economics, Duke University, Durham, North Carolina, United States of America; 5 Nicholas School of the Environment, Duke University, Durham, North Carolina, United States of America; 6 Department of Economics and Resource Management, Norwegian University of Life Sciences, Aas, Norway; 7 Fisheries and Aquaculture Policy and Economics Division, Food and Agricultural Organization of the United Nations, Rome, Italy; 8 Norwegian Seafood Council, Tromsø, Norway; Universidad Veracruzana, Mexico

## Abstract

World food prices hit an all-time high in February 2011 and are still almost two and a half times those of 2000. Although three billion people worldwide use seafood as a key source of animal protein, the Food and Agriculture Organization (FAO) of the United Nations–which compiles prices for other major food categories–has not tracked seafood prices. We fill this gap by developing an index of global seafood prices that can help to understand food crises and may assist in averting them. The fish price index (FPI) relies on trade statistics because seafood is heavily traded internationally, exposing non-traded seafood to price competition from imports and exports. Easily updated trade data can thus proxy for domestic seafood prices that are difficult to observe in many regions and costly to update with global coverage. Calculations of the extent of price competition in different countries support the plausibility of reliance on trade data. Overall, the FPI shows less volatility and fewer price spikes than other food price indices including oils, cereals, and dairy. The FPI generally reflects seafood scarcity, but it can also be separated into indices by production technology, fish species, or region. Splitting FPI into capture fisheries and aquaculture suggests increased scarcity of capture fishery resources in recent years, but also growth in aquaculture that is keeping pace with demand. Regionally, seafood price volatility varies, and some prices are negatively correlated. These patterns hint that regional supply shocks are consequential for seafood prices in spite of the high degree of seafood tradability.

## Introduction

The food crises of 2007–2008 and 2010–2011 demonstrated that sharp increases in food prices can have particularly dire consequences for the poor in developing countries. Poor households often spend more than half of their incomes on food and may even go so far as to take to the streets as a consequence of high food prices [1], [2]. The price of food hit an all-time high in February 2011, and the Food and Agriculture Organization (FAO) of the United Nations’ food price index is currently equal to two and a half its level in 2000 [3]. Some speculate that the current food crisis might have led to the 2011 uprisings in the Middle East and North Africa, as the price of food hit an all-time high in February 2011 [3], [4]. Because high prices ultimately signal shortages in current and expected future food availability [5], tracking price developments is a major tool in understanding poverty and can be an important step in fighting poverty [6].

Until recently, the FAO’s food price index failed to incorporate seafood, a key contributor to the global food system [7].The most comprehensive source of information about global food prices is the FAO’s *Food Outlook* [3], which many governments, nongovernmental organizations (NGOs), and researchers use to analyze the global food system. *Food Outlook* summarizes production and price trends for other major food groups including meat, dairy, cereals, oils and fats, and sugar. The omission of seafood is striking, as fishing dates back at least 40,000 years [8], and seafood now contributes 15% of average animal protein consumption to three billion people worldwide [9]. Moreover, fisheries and aquaculture directly employed 44.9 million people in 2008, with an estimated total of 540 million people deriving their livelihoods from seafood-related industries [9]. A growing supply of fish from aquaculture has allowed higher penetration of seafood in regions where people have traditionally eaten little fish, from inland urban areas to rural areas [9]. The historical and growing importance of seafood suggests that it is time for the international community to consider it as food in global food accounting.

The FAO recently asked our team of researchers to develop a fish price index (FPI) that would fill this important gap in coverage of global food prices. In 2010, preliminary results of this effort were included in a seafood section within *Food Outlook*, but the overall food price index did not yet include seafood [3]. From 2012 on, the FAO intends to incorporate the FPI in its overall food price index. This means that seafood will receive the same coverage the main groups of terrestrial food products have received since 1990 [3]. In this paper, we describe the practical and theoretical challenges in constructing the FPI, justify the methodology and data that were used, and illustrate the uses of the new index.

## Methods

A price index collapses price and quantity information on many different products into a single number. The well-known consumer price index, for instance, attempts to measure the extent to which consumer goods in general in an economy are becoming more or less expensive, where consumer goods represent broad categories like food, housing, gasoline, and healthcare. By analogy, a fish price index (FPI) would track the extent to which seafood as a whole is becoming more or less expensive. The specific challenge in creating the FPI is that it must accurately represent different species and product forms from various regions throughout the world. Even for a relatively homogenous product like wheat, for example, it is unclear what price should be used in constructing a price index. Is it the price in the US or in Ethiopia? Is it the price that a farmer receives at the farm gate or at market? Is it the price of whole grain or wheat flour? For fish, the question is complicated by the diversity of species and the fact that fish is harvested or produced in very different environments, from tropical rivers and lakes via coastal waters to the polar oceans. Moreover, seafood products are sold in a number of different forms with varying levels of processing: whole, filets, croquettes, fresh, frozen, dried, pickled, etc. So for a price index to be useful and relevant, these data must be readily available and easy to update in a timely manner, and the analyst must be able to collapse many product forms and their associated prices into a single index.

Here we justify the specific choices we made in constructing the FPI, including: 1) the choice of a price index formula, 2) the scope of the index in terms of the geographical markets covered and our reliance on trade data, and 3) decisions about which fish and shellfish species and which product forms to include.

### 1. Choice of a Price Index Formula

In price index theory, the main issue is how to represent a large number of prices and quantities with a single price index. In other words, the issue is how to aggregate the many goods in the market into a scalar [10]. For a given time period *t*, 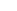, 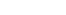 represents the prices of the individual goods and 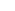, 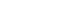, represents the associated quantities. For constructing the FPI, import data are used, meaning that 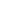
and 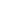
represent seafood import prices and volumes for different importers, species, product forms, and countries of origin.

One approach to aggregation is to assume that the relevant quantities are fixed in a base period (*t* = 0), which gives rise to the Laspeyres index (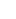):
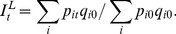
(1)This formulation assumes that the quantities of goods purchased in the base period are relevant for all subsequent periods. It has practical appeal because it is easily updated with price information alone and does not require new quantity data. The standard alternative to (1) is the Paasche index (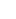) in which the relevant quantities are updated in every period:



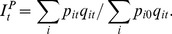
(2)Because the Paasche index updates quantities each period, the index is influenced both by changes in the composition of purchases and the prices. Thus, these indexes in general will arrive at different answers [10]. The conventional wisdom is that Laspeyres will tend to overstate inflation because it fails to account for how consumers adjust their purchasing behavior, for example, in response to new products that exist in today’s market that did not exist twenty years ago. The conventional wisdom for Paasche is that it will tend to understate inflation because it fails to attribute changes in quantities purchased to price changes; a consumer may switch from product A to product B precisely because the price of product A has increased [11].

For the FPI, we adopt the Fisher index (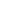), which attempts to compromise between these competing tendencies to overstate and understate inflation. Specifically, the Fisher index is the geometric mean of Laspeyres and Paasche:
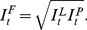
(3)Although it is generally acknowledged that a perfect index formula may not exist, economic theory shows that Fisher is the best index among candidates that are averages of the more intuitive Laspeyres and Paasche indices [10], [12].

Beyond the theoretical rationale, practical considerations in seafood markets also support the use of Fisher. First, in the data that we use for the FPI, updated quantities are available along with prices, so there is no reason to choose the Laspeyres index due to data availability constraints. Second, there are individual examples of seafood markets for which either Laspeyres or Paasche could break down. Technological changes in salmon aquaculture, for instance, have driven down the relative price of salmon and dramatically expanded the salmon market [13], [14]. A Laspeyres index would underweight the importance of salmon in the overall index and make seafood overall seem more expensive than it is. On the other hand, growth in tilapia and pangasius supplies were made possible in part by dramatic reductions in Atlantic cod catches and the resulting market conditions [15]. A Paasche index would fail to account for how rising prices of cod caused substitution into other whitefish species and underweight the importance of cod scarcity.

### 2. Reliance on Trade Data

There are both theoretical and practical reasons that favor relying on trade data from the EU, Japan, and the US for the construction of the FPI. In this section, we argue that the dramatic growth in the global seafood trade exposes the vast majority of the world’s seafood to trade competition. This competition, in turn, suggests that prices from international trade can proxy for non-traded domestic seafood prices. Consider a consumer in a local seafood market choosing between Species A that is traded internationally and Species B that is not traded internationally. Because the seller of Species A in the local market could alternatively sell the product into the global market, the global market price will affect the price charged in the local market. But because Species B is on sale next to Species A, price of Species B will not be independent of the price of Species A. Thus, trade in Species A will influence the price of Species B even if Species B is never traded internationally. Use of trade data has the added virtue that it can be updated easily. The particular choice of data from the EU, Japan, and the U.S. is reasonable because these regions account for a large fraction of total seafood imports globally, these regions import seafood from countries throughout the industrialized and developing worlds, and these regions produce high quality data that is readily available and updated regularly.

**Figure 1:**
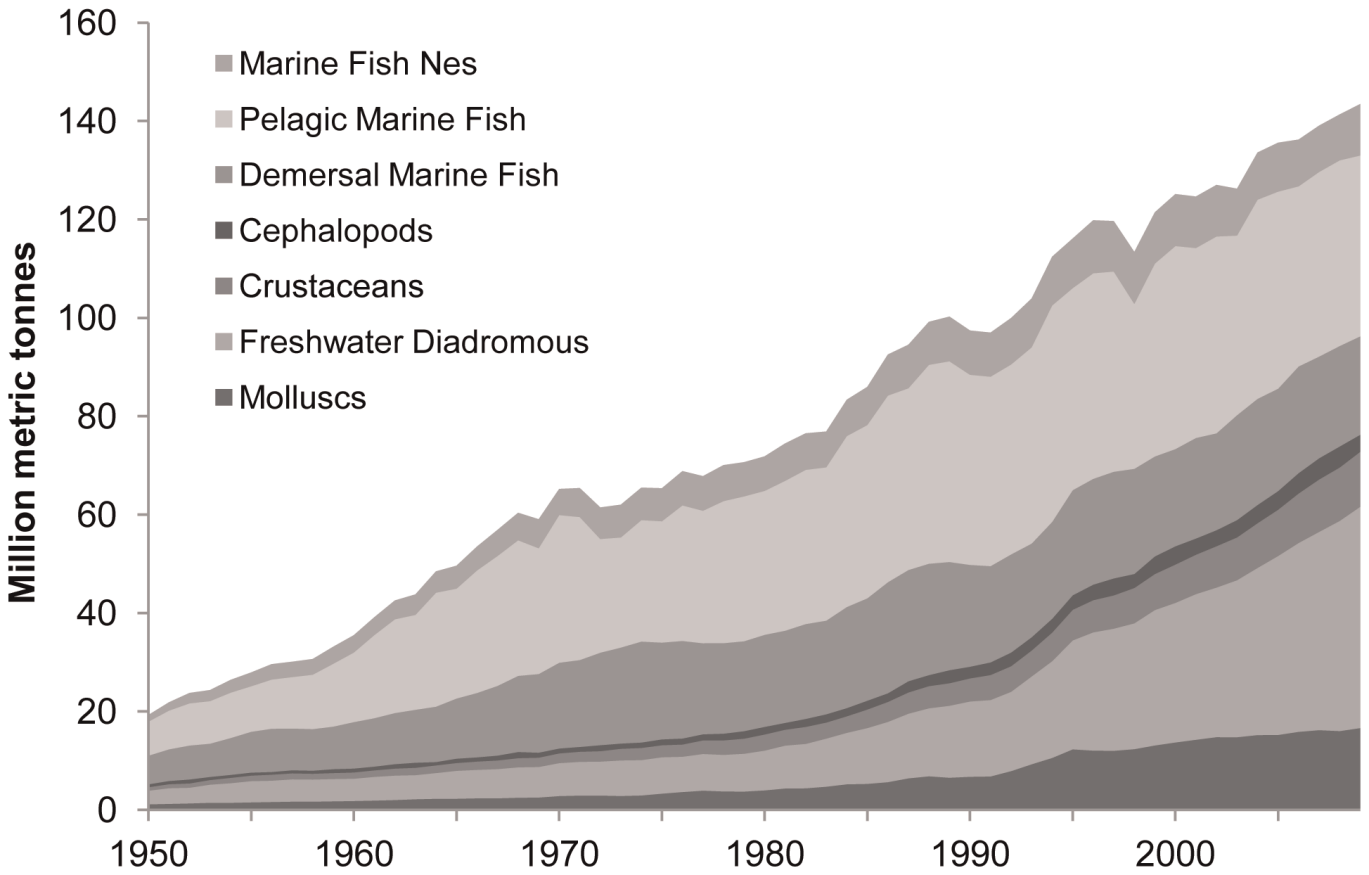
Global seafood production 1950–2009.

The global seafood trade has expanded dramatically in the past 60 years. The supply of all major fish and seafood species has increased (Figure 1), and overall global seafood production grew 3.5% annually during this period [16].

Trade of fish and seafood products has increased alongside growth in global seafood production. Seafood has long been among the most internationally traded food products [17]. Thirty-nine percent of all seafood production in 2008 was traded internationally [9]. A number of factors have caused the increased trade in seafood. Transportation, freezing and cooling technologies, and logistics have improved significantly, reducing transportation costs and opening up the trade of new product forms such as fresh seafood [17]. Growth of supermarket retail chains and aquaculture are also key contributors to the booming international seafood trade [9], [18]. Trade, in turn, has increased competition in international seafood markets, leading to more market integration for both traded and non-traded seafood products [18]. The supermarket revolution ultimately facilitates international price competition. Supermarkets now reach many urban poor in developing countries [19], and supermarket procurement practices in particular ensure that domestic fish is meeting increasing levels of competition from internationally traded fish [19], [20].

We now quantify the extent of trade competition for seafood in various countries. To estimate the extent of exposure to trade competition for a given commodity in a given market, the first step is to compute total exports and imports relative to total domestic consumption. The intuition is that exports and imports measure the absolute amount of trading activity. When this amount is high compared to domestic consumption of the product, then trade is an important influence on domestic consumption. Although the ratio does not inform how domestic markets are exposed to trade competition, it provides an indication of the extent of trade competition. Similar measures are used in macroeconomics to proxy for openness of the economy [21].

To estimate seafood trade competition, we use FAO seafood consumption statistics for 2005 [16], the latest year with complete information available. The database contains seafood consumption in 223 countries and territories. Consumption is defined as domestic production with the addition of imports and the subtraction of exports. Global consumption for 2005 was 135 million tons, of which 107 million tons were for human consumption. Twenty-four out of the 223 countries did not report any seafood trade, while one country, Luxembourg, did not report any seafood production.

**Figure 2:**
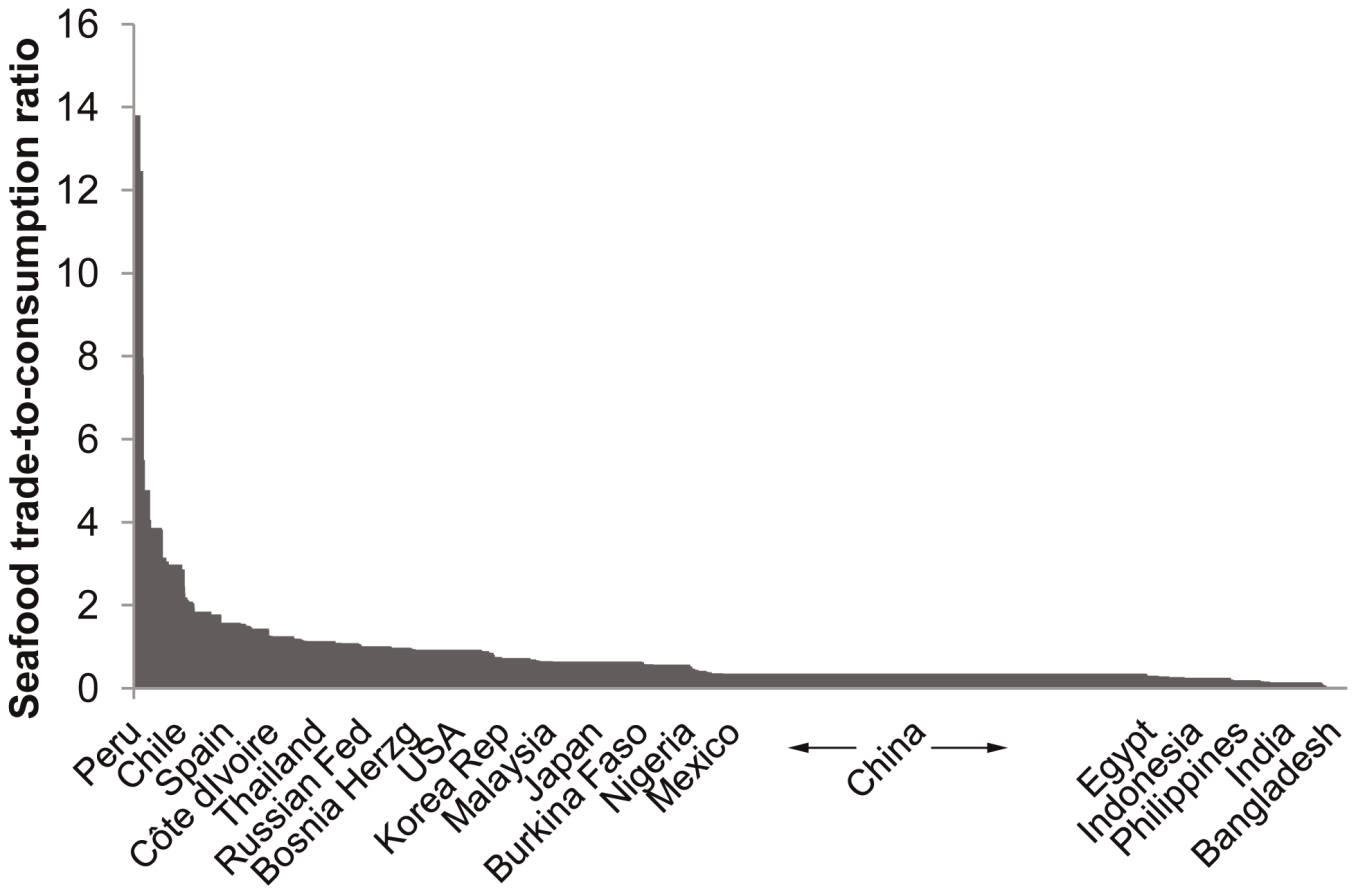
The seafood trade to consumption ratio for 220 countries (Iceland, Faroe Islands and the Falklands omitted).

Figure 2 plots the ratio of exports plus imports to seafood consumption weighted by total consumption for all countries and territories. Countries are ordered from largest to smallest ratios, and the width of each bar is the weighting for the size of the country’s domestic consumption. Hence, China has the widest bar and is depicted with arrows on either side to illustrate its expanse. Due to space limitations only some of the 220 included countries and territories are explicitly named on the horizontal axis. Also note that Iceland, the Faroe Islands and the Falklands are excluded from figure 2 to improve readability because their ratios are so high that they completely overshadow the magnitudes of other countries’ ratios. Iceland has the highest ratio at 39.9 because most of the landings are exported and there is limited domestic consumption. That is, Icelandic exports plus imports of seafood products are nearly 40 times the level of domestic seafood consumption. Similarly, the Faroe Islands have a ratio of 26.0, and the Falklands have a ratio of 20.3.

There are many countries (82) for which the trade ratio exceeds 1. In these countries, a significant share of domestic landings is exported and/or a significant share of consumption is imported. For example, the US–which in 2005 was the world’s third largest seafood consumer, the fifth largest producer, the fourth largest exporter and the third largest importer– has a ratio of 0.926. A key driver of this ratio is that four of the leading species in US consumption are primarily imported (shrimp, tuna, salmon and tilapia), and only one (Alaska Pollock) is primarily sourced from domestic landings [18]. China has a ratio of 0.346 and is the most important nation in all categories–except for exports where it is second only after Peru. The interpretation is that the quantities of seafood imported and exported in China correspond to 34.6% of the seafood consumed domestically.

Even with a ratio above one, analysts cannot be sure that all of the seafood in a country is exposed to trade competition. There can be species with no tradable substitutes or, in extreme cases, some rural or coastal areas that are completely isolated from the international seafood trade. In such cases, the ratio is likely to overestimate trade exposure. Still, in countries with a high ratio, a very large share of the seafood consumed will be exposed to trade competition. Similarly, in a market with a very low ratio, this ratio is likely to underestimate trade exposure, as many domestically consumed fish that are not traded are exposed to trade competition, especially in densely populated urban areas or in regions with major ports.

To estimate how much seafood is exposed to international trade, we define a threshold ratio; for ratios above the threshold we assume that all seafood in that country is exposed to trade competition, and for ratios below it we assume that only the seafood that is actually exported or imported is exposed to trade competition. Because there is standard way to define a threshold like this one, we explore how different assumptions about the threshold yield different estimates of exposure to international trade.

Table 1 reports the cumulative share of global seafood consumption associated with different thresholds of the trade ratio. For a threshold at 0.25, 88.7% of the seafood consumption is exposed to trade competition.

**Table 1:** Trade to consumption ratio and cumulative share of food fish consumption.

Trade/Consumption Ratio (%)	Low						High
	0.1	0.2	0.25	0.3	0.4	0.5	1
Cumulative share of seafood exposed to trade (%)	98.0	91.8	88.7	87.1	62.8	62.2	53.0
With special treatment for China (%)	86.9	80.8	77.7	76.1	73.9	73.2	64.1

To explore the implications of our assumptions further, we also separate out China in Table 1. More than 86% of the seafood consumption is exposed to trade up to a ratio of 0.34, but the percentage moves down to 64.5% at a ratio of 0.35 because China’s ratio is at 0.346. China is such a large consumer, with 31% of the total global consumption, and such a large trading nation that the estimation method for China makes a significant difference in the global share of seafood exposed to trade competition. It is difficult to justify putting China on either side of the threshold because China produces large volumes of fish that are almost certainly exposed to trade competition, especially products like tilapia and shrimp that are major exports. China is the world’s leading tilapia producer, and about half the production is exported [9]. At the same time, there are large volumes of fish that most likely are not (or only minimally) exposed to trade competition, especially production of grass carps in rural areas. A large share of the production of grass carp is consumed locally in rural areas where there is limited trade competition, but one can also find grass carp at markets and restaurants in the cities being exposed to trade competition. Supposing that 50% of China’s seafood consumption is exposed to trade competition–an assumption we believe is conservative–and assuming an otherwise conservative threshold of 0.25, we estimate that 77.7% of the world’s seafood consumption is exposed to trade. Moreover, the second row in table 1 indicates that the share of seafood consumption that is exposed to trade competition becomes much more stable when China is given this special treatment, as it varies between 73.2% at a ratio of 0.5 to 86.9% with a ratio of 0.1.

Clearly, a significantly higher share of the world’s seafood consumption is exposed to trade competition than the 39% that is actually traded. With different assumptions, one can assert that the share of seafood consumption that is exposed to trade competition is somewhere between two thirds and four fifths of the total consumption, with most of the uncertainty depending on how China is treated.

While the arguments above justify the reliance on trade data in general for the FPI, here we argue that the particular choice of data from the EU, US, and Japan is practical. All three regions have well-developed and easily accessible trade data systems. Thus, it is possible to update the index regularly with both import prices and import quantities. These regions are also highly representative of the global seafood trade. More than 80% of the world’s seafood imports (in terms of value) are into developed countries [16]. Most countries that export fish products ship to one or more of these three regions, and these regions represent around 90% of the total developed country imports.

### 3. Choice of Species and Product Forms

Here we explain why data on fresh and frozen white fish, salmon, crustaceans, pelagic fish excluding tuna, tuna, and a broad category of other fish can represent global seafood prices. The main reason is that these categories account for roughly two thirds of seafood imports (measured in value) into the EU, Japan, and the US and more than half of all imports globally. Moreover, an analysis of pairwise price correlations over time reveals a pattern of increasing price convergence among species groups. This pattern supports the use of these main species categories as representative of the global seafood trade in general.

Table 2 details the species included in our main species group: white fish, salmonides, crustaceans, pelagic (excluding tuna), tuna, and other fish. We include only fresh and frozen seafood in these broad categories. That means our index excludes highly processed products like fish sticks and soups. The rationale for this decision is that these other product forms have many inputs (e.g. cereals, oils, spices, vegetables, etc.) in addition to fish, and we do not want the FPI to reflect price movements in these other foods. Also, our index excludes some fish and shellfish species not captured by the broad categories in Table 2.

**Table 2:** Composition of seafood species groups.

Group	Species
**White fish**	Anglerfish, catfish, codfish, flounder, Greenland halibut, haddock, hake, halibut, hoki/blue grenadier, ling, megrim, Nile perch, other flatfish, other whitefish, pangasius, pink cusk-eel, plaice, pollack, Ray’s bream, redfish, saithe, sea bass, sea bream, sole, tilapia, whiting, wolffish
**Salmonidae**	Atlantic salmon, chum, coho, other salmonidae, other trout, pink, rainbow trout, sockeye
**Crustaceans**	Crab, crawfish/rock lobster, crayfish, edible crab, krill, Lobster, Norway lobster, other crustaceans, prawns
**Pelagic excl. tuna**	Anchovy, blue whiting, brisling/sprat, capelin, herring, horse mackerel, mackerel, sardine/sardinella, Southern blue whiting
**Tuna**	Albacore, bigeye tunas, bonito, bluefin tunas, other little tunas, other tunas, plain bonito, skipjack/stripe-bellied bonito, Southern bluefin tunas, yellowfin tunas
**Other fish**	Alfonsino, amberjack, ayu sweetfish, barracudas, bass/perch freshwater, butterfish, croaker, dolphinfish, eel, hairtail, marlin, mullet, other fish, other freshwater fish, other saltwater fish, other sharks, pike/pickerel, pufferfish, sauger, smelt, snapper, sturgeon, swordfish, toothfish, yellow perch

The trade data from the EU, US, and Japan for species in Table 2 are highly representative of global seafood markets. For 2010, the most recent year with complete data, fresh and frozen seafood from categories in Table 2 account for 70%, 75% and 63% of total seafood import value for the EU, the US, and Japan respectively. Thus, in spite of excluding some species and product forms, the data capture roughly two thirds of all seafood imports into these major markets for a total of over $47.5 billion in value in 2010 (Table 3). Moreover, these three regions account for 52% of total seafood imports globally when measured in value for the year 2008, the last year for which the FAO reports global seafood trade figures [16]. Table 3 also shows how the composition of import value for different species groups varies across the three regions. The total column provides an indication of the relative importance that each species group will have in constructing the FPI.

**Table 3:** Import value in million USD in 2010 for major seafood groups in the EU, the USA, and Japan.

	EU15		Japan		USA		TOTAL	
	*Million USD*	*%*	*Million USD*	*%*	*Million USD*	*%*	*Million USD*	*%*
**White fish**	5,818	28%	1,439	11%	2,398	17%	9,655	20%
**Salmonidae**	4,887	24%	1,824	15%	2,018	14%	8,729	18%
**Crustaceans**	4,778	23%	3,484	28%	6,848	47%	15,110	32%
**Pelagic excl. tuna**	2,286	11%	614	5%	240	2%	3,140	7%
**Tuna**	2,267	11%	2,176	17%	1,404	10%	5,846	12%
**Other fish**	1,907	9%	3,018	24%	1,542	11%	6,467	14%
**TOTAL**	20,506	100%	12,554	100%	14,449	100%	47,509	100%

To move from import values to prices, we divide import value by import volume for each month. This operation is done at the most disaggregated level of the data available. With different product forms (fresh whole, fresh fillet, frozen whole, frozen fillet, frozen “surimi” or fish paste, frozen fish meat, chilled whole, chilled fillet), production technologies (aquaculture and capture), import markets (the EU, the US, and Japan), and exporters (Africa, Asia, Europe, North America, Oceania, and South America), there are a total of 608 seafood import categories. Because import prices are reported in each region’s currency (i.e., euros, dollars, and yen), we convert all prices to USD using nominal exchange rates. These calculations implicitly assume complete exchange rate pass through – defined as “the percentage change in local currency import prices resulting from a one percent change in the exchange rate between the exporting and importing countries” [22]. Empirical evidence of exchange rate pass through for seafood supports this assumption [23], [24].

We now analyze these individual price series to evaluate our assumption that, given high degrees of trade competition, import prices are likely to reflect seafood prices for non-traded products. To this end, we ask whether price movements across different seafood products are becoming more aligned. We test this working hypothesis in a simple way by comparing price correlations across a range of seafood products. We create sub-indices based data used for five species groups: white fish, salmon, shrimp, pelagic without tuna, and tuna with origins from Africa, Asia, Europe, North America, Oceania, and South America. We have a total of 28 price indices (five species groups by six continents for a total of 30 prices indices, but since neither Africa nor Asia have any significant salmon exports, the final number of prices was 28). Each price series was divided into two, with one part covering January 1990 to December 1999 and the other January 2000 to December 2010. Then pairwise price correlations were calculated, producing 392 correlation coefficients for each of the two periods. The underlying hypothesis is that markets have become more integrated over time, and thus comparing the 1990s with the 2000s we should see more positive correlations in prices.

**Figure 3:**
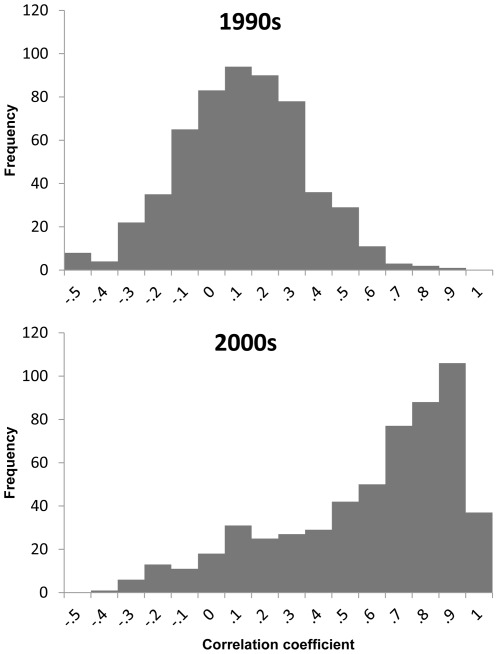
Price correlation among 28 seafood import categories during the 1990s and 2000s.

The results a pattern that is consistent with price convergence and an increasing importance of trade competition. Figure 3 shows the distribution of the 392 price correlation coefficients as histograms for the two sample periods. The distribution for 1990s has a mean of 0.06 and a standard deviation of 0.23 compared with 0.53 and 0.32 for the 2000s. Thus, during the 1990s, on average, prices across species groups and markets did not tend to follow the same trend in contrast to the 2000s, when price movements have become more uniform with an average correlation of 0.53.

This discussion also reinforces the value in constructing a global seafood price index, as trade competition is integrating seafood markets worldwide. Moreover, it suggests that a seafood price index is not only relevant for traded products but also for non-traded seafood.

### 4. Constructing the Index

The FPI is calculated and updated based on the 608 unique trade data categories of fish and seafood described above. The individual categories allow us to aggregate subsets of the individual items to create disaggregated fish price sub-indices. For example, we construct separate price indices for capture and farmed fish, which respectively account for 70% and 30% of the 608 categories. For each month, we compute import prices for each category and covert to USD as described above. We then compute the index itself using equations (1)–(3). Fluctuations in fish catches and aquaculture harvests lead to uneven export flows and occasional months during which there is no trade for certain categories. Given these fluctuations, to avoid a base value of zero, we use a base period that spans several years. Specifically, we use the average value over the period 2002–2004 as our base, which is the standard for FAO food price indices [25]. Finally, note that the trade statistics used for constructing the index is provided by the Norwegian Seafood Council in cooperation with the FAO.

## Results

**Figure 4:**
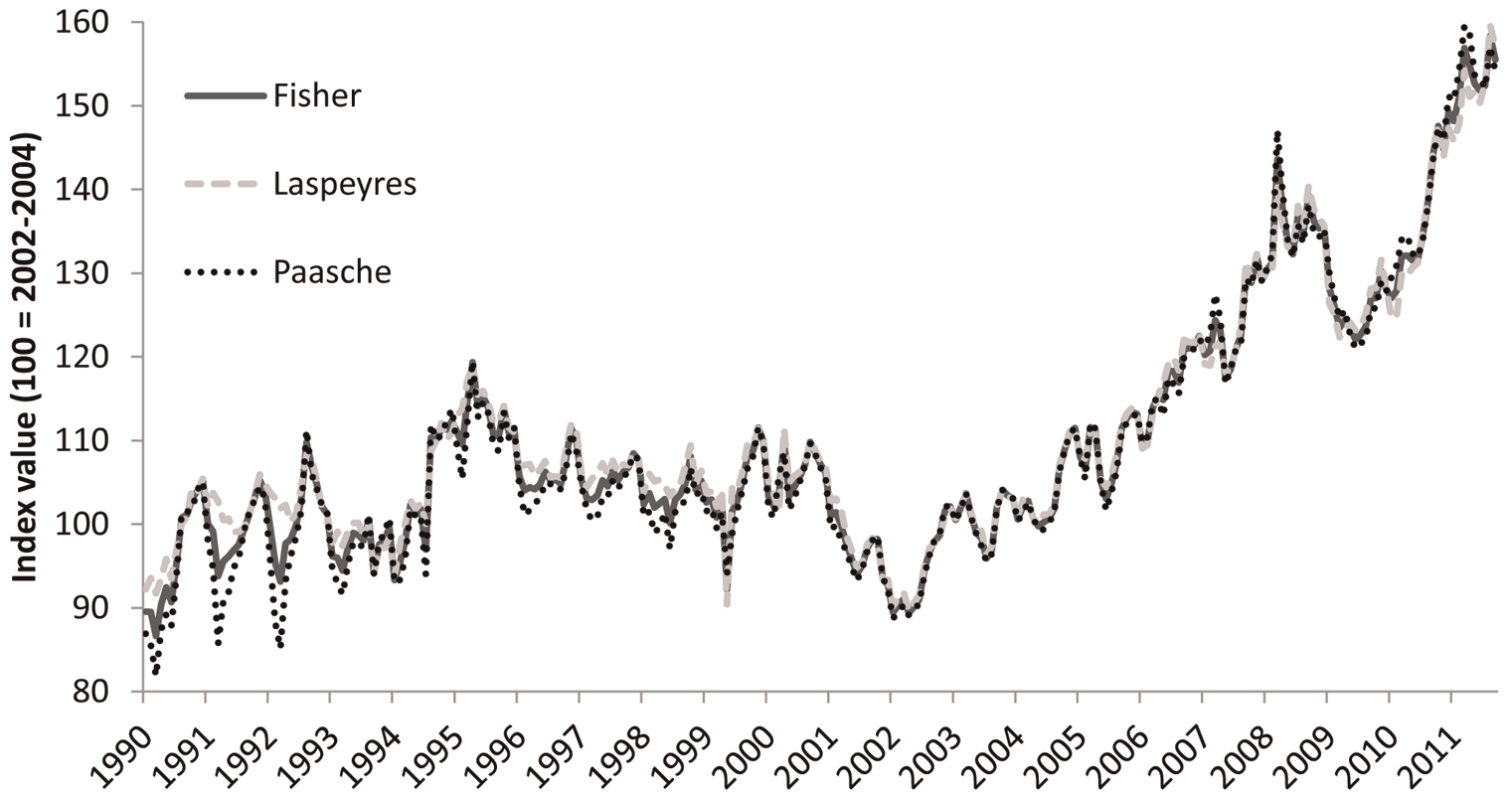
The FPI calculated using Fisher, Laspeyres and Paasche formulas.

In the Methods section above, we explain why we chose the Fisher index for the FPI. It turns out that the two component indices – Laspeyres and Paasche–show comparable results. Figure 4 depicts the three indices together. With few exceptions, all three indices reflect similar price movements. This is not surprising as in a time series context the difference between Laspeyres and Paasche from one period to next is usually small [10]. Moreover, these results reflect that price movements of different seafood products have become more aligned. As a result, the FPI depends less on the particular weighting scheme used for aggregating import prices. In contrast, seafood prices during the early 1990s are less correlated (see figure 3) and the corresponding FPI values are further away from base period (100 = 2002–2004), which is why deviations between Laspeyres and Paasche are larger.

**Figure 5:**
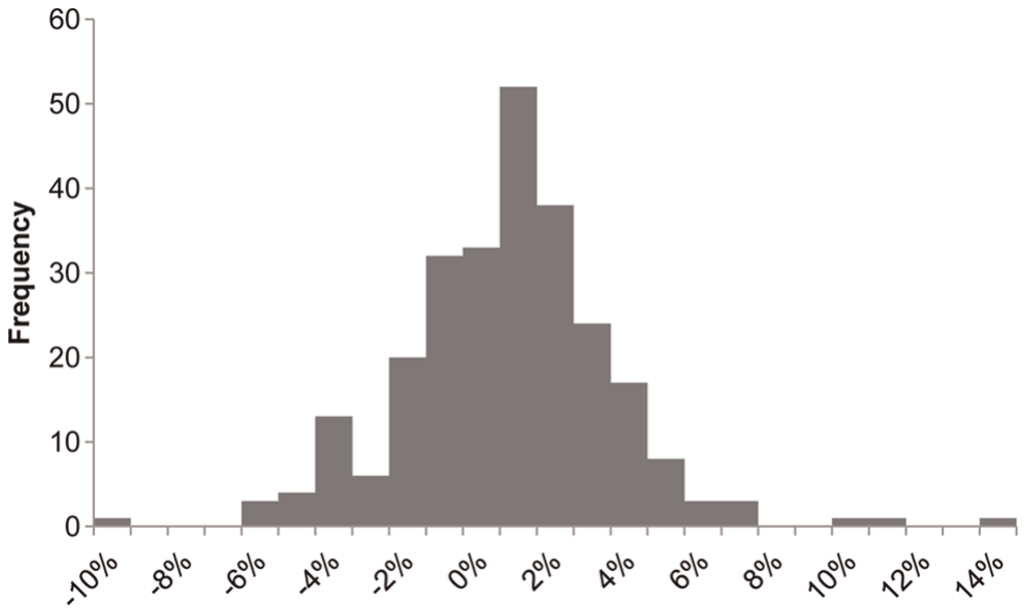
Distribution of monthly changes in the FPI.

We can further explore the behavior of the FPI by computing monthly percentage changes. The average monthly change from January 1990 to July 2011 is 0.2% and with a standard deviation of 2.8%. Figure 5 depicts the distribution of these changes. Not surprisingly, the FPI changes appear to exhibit normality. After all, FPI changes are averaging price changes for 608 underlying categories, suggesting the relevance of the Central Limit Theorem. Still, there are some apparent outliers that could reflect major disruptions in seafood markets.

**Figure 6:**
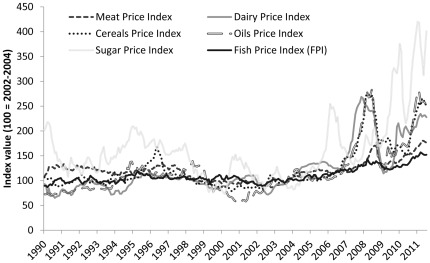
The FPI together with the traditional FAO food price indices.

Figure 6 shows the FPI along with the FAO’s other food price indices for meat, dairy, cereals, oils and sugar. Compared to prices of terrestrial food, fish prices appear to be less volatile. An important corollary to the greater relative stability of the fish price index is that fish prices appear less subject to price spikes, such as the 2008 and 2011 price spikes in the prices of cereals, dairy, and oils. A comparison of price development during the 1990s and the 2000s helps to demonstrate the differences in trends and volatility. From 1991 to 2000 the FPI rose 6.6% while the food price index fell 12.8%. In the following decade from 2001 to 2010 both indices rose, but the 40.9% growth in fish prices was less than half of the increase in food prices, which rose by 98.3%. Thus, in a period when food prices were declining, fish prices experienced a slight increase, and then after the turn of the millennium, as food prices spiked to record highs, fish prices grew at a comparatively moderate pace. The standard deviation of price changes during these two decades was 5.6% for the FPI and 12.2% for the food price index. This implies that terrestrial foods have a volatility that is twice as high as that for fish.

Meat appears to have a price development more similar to that of fish than other terrestrial food. During the two decades represented by the 1990s and the 2000s, meat prices fell and rose with −23.6% and 57.8% and had standard deviations of 9.7%. While this indicates that meat experience higher price volatility than fish, it is nevertheless significantly less than other terrestrial food. It is interesting to note that as the global supply of seafood has become more influenced by aquaculture, fish prices have become more competitive relative to meat prices. The role of aquaculture in the formation of seafood prices is one of the issues that we will explore more in what follows.

The FPI can also be disaggregated into different sub-indices. This process allows us to investigate how seafood markets in different countries and regions are linked, and how prices for different species and product forms influence each other. We provide two examples related to important policy issues: the relationship between wild and farmed fish, and the extent to which the market is global.

The most significant change in the global fish production during the previous four decades has been the growth in aquaculture, which for decades has been the world’s fastest growing food production technology. The merits of aquaculture are debated, however. For some, aquaculture is regarded as a highly promising food production technology that is already fulfilling some of its potential [9], [14], [26]. For others, aquaculture is regarded as an environmentally degrading production technology increasing production using unsustainable practices with clear limits to how much can be produced [27].

**Figure 7:**
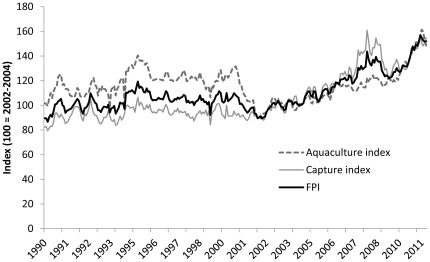
The FAO fish price index, the aquaculture index and the capture index.

Figure 7 shows the aggregated FPI as well as the index disaggregated for wild and farmed fish between 1994 and 2010 using the mean in the 2002–2004 period as base. Price movements for wild and farmed fish are similar through the 1990s, but prices diverge at the turn of the century. The price index for farmed fish declines, and it subsequently has only a weak positive trend. The index for wild fish starts increasing in 2002, and although limited compared to other foodstuffs, it shows an impact of the food crisis in 2007–2008, as it is peaking in 2008. In contrast, the food crisis of 2007–2008 had little visible impact on the aquaculture index. These trends suggest that the increasing supply of fish from aquaculture has kept pace with demand as a consequence of innovations that have reduced production cost [14].

Prices also suggest that the growth of aquaculture has not been constrained by its use of wild fish resources. In the market for wild fish, where aggregate production cannot increase to any significant extent, increasing demand can only increase prices. Substitution will dampen price pressure as the demand spills over to farmed fish. For salmon this effect is obvious, but for tuna and other high-value wild species that have little to no substitutes from aquaculture, the substitution effect is less pronounced [28]. Because of these market interactions, one would not expect the price indices for capture and aquaculture fish to continue to drift apart.

**Figure 8:**
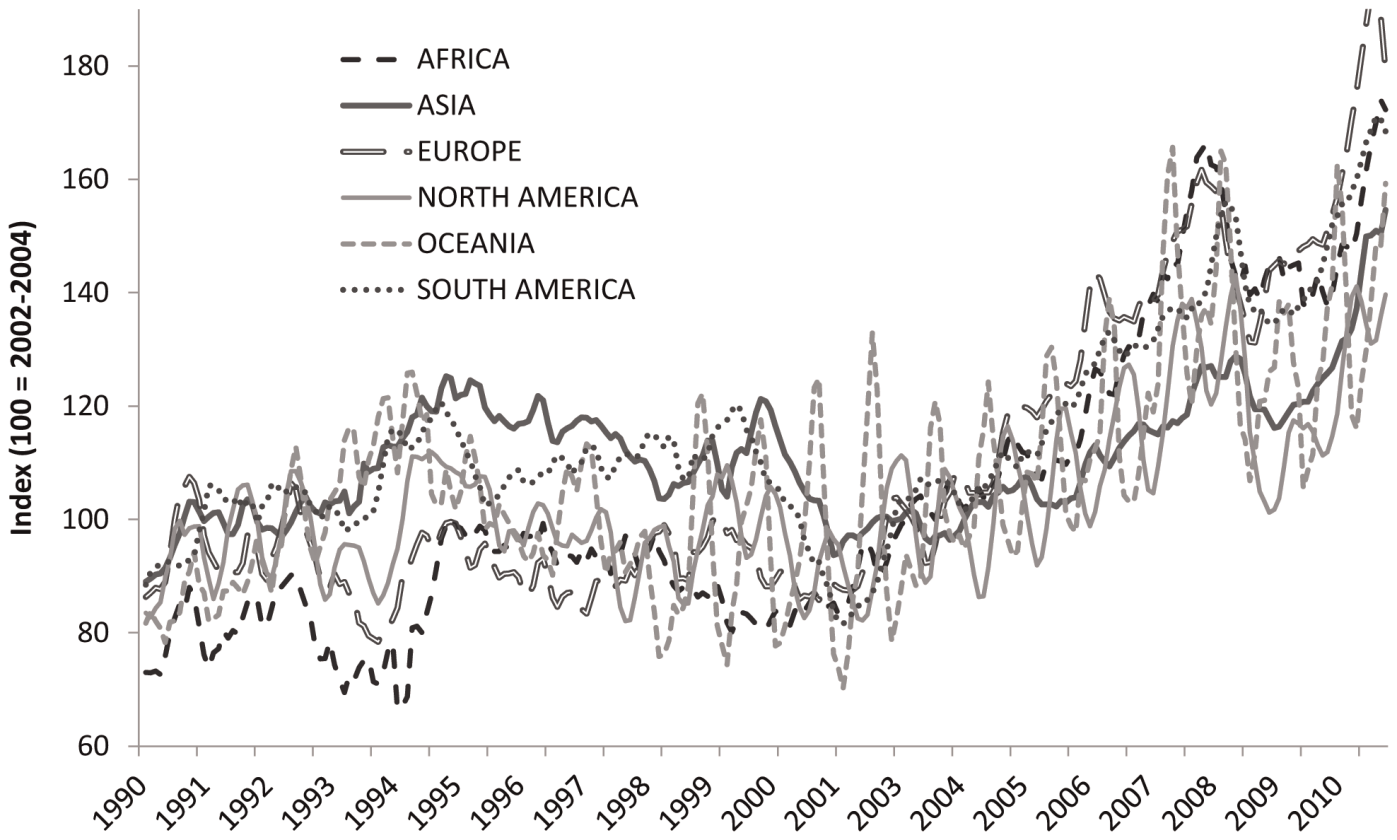
The FAO fish price index by continent (smoothed with a three-month centered moving average).

In Figure 8, the FPI is shown by region. The main trend is similar for all regions, suggesting a globally integrated market despite the fact that the fish species that make up the price index vary considerably across regions. The impact of aquaculture can most clearly be seen for Asia, the region where most of the world’s aquaculture production takes place. Price increases have been less pronounced in Asia compared to other regions because lower prices in aquaculture have dampened upward pressure on regional seafood prices. Of equal importance is the fact that the greater control of the production process allowed by aquaculture and the resulting more stable supply of fish significantly reduce price volatility, which is beneficial to both fishing and fish-farming households [29].

## Discussion

The FPI we developed provides a new tool to understand global seafood markets and can be used to help inform global food policy. The FPI is an important addition to the FAO’s food price information, which is itself used by a number of governments, NGOs, and researchers worldwide. The fact that fish prices do not appear to track any terrestrial food groups perfectly reinforces the need for the FPI. Incorporating the FPI into FAO’s food price index will help to provide a more comprehensive picture of world food prices.

With the increased importance of fish consumption worldwide, uncertainty over the future supply of fish makes the introduction of the FPI even more pertinent. Overfishing [30], climate change [31], and the dependence of aquaculture on capture fisheries [27] are issues that threaten to destabilize – if not decrease – global fish supply. Moreover, the species composition of fish catches has changed over the past decades, and researchers debate the extent to which these changes signal a pattern of sequential overexploitation and degradation of marine ecosystems [32], [33]. In terms of the aggregate fish supply, however, the growth in aquaculture has more than compensated for stagnating supply from capture fisheries, enough to make per capita consumption of seafood to continue to increase, reaching a record level of 17 kg per capita in 2008 [9]. This development has been possible due to dramatic changes in the seafood market.

The FPI can contribute to sustainable development by providing early warning signals about rising seafood prices that have the potential to affect many people throughout the world who depend on affordable fish protein. Tracking price changes in the FPI and comparing them to price changes for others foods may shed light on how seriously these issues are impinging on global food supplies. If the FPI starts to trend upward more than prices of other animal protein sources, it may reflect unsolved problems of overfishing, aggregate impacts of climate change, reaching the limits of forage fish used in aquaculture feed, or other degradation of marine ecosystems. Alternatively, an FPI that continues to trend lower than other animal proteins could indicate improvements in oceans governance, net positive impacts from climate change, or continued technological change in aquaculture that lowers costs and ultimately prices to consumers.
